# Calling for justice with #JusticeforBreonnaTaylor: a case study of hashtag activism in the evolution of the black lives matter movement

**DOI:** 10.1007/s13278-023-01054-3

**Published:** 2023-04-08

**Authors:** Miyoung Chong

**Affiliations:** Department of Journalism and Digital Communication, University of South Florida, 140 7th Avenue South, St. Petersburg, FL 33701 USA

**Keywords:** Twitter, Social network analysis, Hashtag activism, Breonna Taylor, Black lives matter, Natural language processing, Black Twitter

## Abstract

Taking a stage-based approach, before and after the release of the 15-h audio recording files of the grand jury’s inquiry on the Breonna Taylor case on October 2, 2020, this study examined the #JusticeforBreonnaTaylor Twitter networks. By employing multimethodology, including natural language processing, social network analysis, and qualitative textual analysis, I examined keys connectors of the two Twitter networks and investigated major themes conducting thematic analysis of network discourses and highly associated hashtags with the hashtag #JusticeforBreonnaTaylor. In both networks, several key stakeholders, such as Benjamin Crump, Danial Cameron, and Black women activists were identified as key connectors along with social activists and ordinary participants. Demanding justice to the case was the core agenda of the hashtag activism. The findings of the study revealed that the participants not only shared breaking news and important information but also organized protests and routinely tagged people to spread messages about the Taylor’s case on Twitter. The participants conversed major issues about the Taylor case and set the agendas for the next action, such as encouraging to take part in voting for the 2020 presidential election. The thematic analysis concurrently demonstrated that the network participants strongly demanded legal prosecution to the three Louisville cops that involved in the act of killing Breonna Taylor during the botched raid in her apartment.

## Introduction

Breonna Taylor, a Black woman and 26-year-old emergency room technician, was killed in the bedroom at her apartment on March 13, 2020, during a botched raid by the Louisville police. George Floyd’s death on May 25, 2020, made public attention revisited to the case of Breonna Taylor’s murder. Twitter was heated by calling for justice to the cops involved with extrajudicial killing of George Floyd and Breonna Taylor. As a continuation of the Black Lives Matter movement, #JusticeforBreonnaTaylor and #JusticeforGeorgeFloyd along with #Ragethedegree became the top trending hashtags in the USA in 2020 summer.

Hashtag activism was described as the “acting of fighting for or supporting a cause with the use of hashtags as the primary channel to raise awareness of an issue encourage debate via social media” (Tombleson and Wolf 2017, p. 15). Burgeoning literature centered on hashtag activism has revealed that hashtags functioned as organizing instruments or ideological weapons to disseminate ideas and create conversations affiliating rather fragmented individuals with online communities by facilitating Internet resources in digital platforms (Bonilla and Rosa, 2015; Chong 2019b; Yang 2016). Some scholars indicated that via framing process integrated by digital platforms, such as social media, activists and grass root organizations could shape public agendas and attitudes regarding a specific issue (Chong 2019b; Hallahan 1999; Hon 2016). The death of Breonna Taylor emerged as one of the most prominent case studies of hashtag activism and epitomized the Black Lives Matter Movement centered around a young Black woman.

This study applied a stage-based approach, which are before and after the conclusion of the criminal proceedings against the police officers involved in Taylor’s death and the release of the audio recordings of the grand jury’s inquiry. By employing multimethodology, including natural language processing, social network analysis, and qualitative textual analysis, I examined keys connectors of the two phases of the #JusticeforBreonnaTalyor Twitter networks and investigated major themes through the thematic analysis of network discourse and highly associated hashtags with #JusticeforBreonnaTaylor.

## Literature review

### The rise of digital community and social media activism

In *Democracy in America*, Tocqueville praised the America’s communal attribute as the entry to American democracy. In 19th-century New England, as Tocqueville reported, self-governing communities where citizens enjoyed the bureaucratic functions that they felt compelled to play. American citizens had ample opportunities to be engaged in politics in a substantial manner while nurturing the community members to trust in public affairs and educating them to conform their lives to a larger society (Bellah et al. 1996; Tocqueville 2012).

However, in the digitalized and globalized society, the meanings of “community” and “interactions” were not only reconceptualized due to the Internet revolution that harvested online communities but also provided ample opportunities in innovate ways of socio-political engagement, especially via social media and social networking platforms (Allen and Light 2015; Chong 2018; Hargittai and Shaw 2013). Particularly, social media enabled citizens to convene and mobilize to go after real-world changes based on shared civic goals and values (Rosenberry, 2010).

In July 2013, activists Alicia Garza, Patrisse Cullors, and Opal Tometi created the hashtag #BlackLivesMatter after George Zimmerman’s acquittal for his second-degree murder charge on Trayvon Martin’s death. The Twitter hashtag was unpopular and shared only a few hundred times for more than a year until August 2014. The Black Lives Matter movement regained national attention, especially following the deaths of Michael Brown and Eric Garner in 2014. On August 9, 2014, Michael Brown, an 18-year-old unarmed African-American man, was shot by a white police officer in Ferguson, Missouri. The Twitter hashtags, #Ferguson, #MichaelBrown, and #IfTheyGunnedMeDown, were created to protest police violence caused his death. Before a week passed after Michael’s death, millions of Twitter users shared the hashtags #Ferguson (21,626,901 times), #MichaelBrown/#MikeBrown (9,360,239 times), and #BlackLivesMatter (4,312,599) (Bonila and Rosa 2015; Freelon et al. 2016, p. 21).

The movement on social media using #BlackLivesMatter or #BLM called attention to the deaths of unarmed Black people by state-sanctioned violence at large and provoked unceasing protests in many cities across the USA and online communities (Freelon et al. 2016; Kang 2015, May 4). During the summer of 2015, Black Lives Matter activism engaged in the 2016 USA presidential election campaign by demanding policy proposals to focus on black people’s deaths in police custody (Eligon 2015, November 19; Resnikoff 2015, July 18). Since the Ferguson outcries, #BlackLivesMatter participants protested against police brutality and violence against Black individuals, including Alton Sterling (July 5, 2016 in Baton Rouge), Philando Castile (July 6, 2016 in Falcon Heights), Sylville Smith (August 13, 2016 in Milwaukee), Terence Crutcher (September 16, 2016 in Tulsa), and Keith Lamont Scott (September 20, 2016 in Charlotte), among others. The deaths resulting from police violence reinvigorated the Black Lives Matter movement across the USA while calling for a civil rights investigation to the US Department of Justice.

Social media was adopted to nationally disseminate Michael Brown’s story. Without counting on traditional news media platforms, activists and supporters could spread their agendas and narratives on Twitter, which coined the concept of Black Twitter. Jones said, “Black Twitter can be described as a collective of active, primarily African-American Twitter users who have created a virtual community that participates in continuous real-time conversations. When they work together, this collective is proving adept at bringing about a wide range of sociopolitical changes” (Jones 2013, July 18, p.6). Claiming social media’s significant contribution to the success of the Black Lives Matters (BLM) movement, a journalist stated, “If you’re a civil rights activist in 2015 and you need to get some news out, your first move is to choose a[n online] platform,” (Stephen 2015, October).

The vast majority of BLM participants on Twitter tenaciously condemned police brutality and demanded justice for the victims (Freelon et al. 2016). However, convicted white policemen participated Black people’s death were often exonerated and allowed to back to the duties without proper legal punishments. A former Minneapolis police officer, Derek Chauvin’s 20 years sentence for murdering George Floyd was the first conviction of a white officer in Minnesota for the murder of a Black person. The trial, held from March 8, 2021, through April 20, 2021, was the focus of media coverage, and more than 23 million people watched the ruling on live. Breonna Taylor was killed around two months before George Floyd but the historic conviction and verdict to the Minneapolis police officers on George Floyd’s case brought the public’s attention to the court’s decision on Breonna Taylor’s case.

Despite growing media spotlight, the BLM protest has gained little academic attention, and there is a dearth of empirical research on the BLM movement (Updegrove et al. 2020). In particular, social media activism scholars mainly investigated the features of connective and networked relationships and leading groups enabled by Web 2.0 technology (Gerbaudo 2018). Concentrating on socio-political aspects and historical contexts, BLM social media scholars employed either descriptive analysis, survey methods with quantifying manners or ethnographic or textual analysis with qualifying manners (Clark 2019; Clayton 2018; Freelon et al. 2016; Updegrove et al. 2020). However, BLM researchers have yet fully described why and how some individuals and institutions play pivotal roles in the movement. Moreover, a data science approach for examining the BLM movement is in the beginning stage.

### From #OscarGrant to #TrayvonMartin

**Fig. 1:**
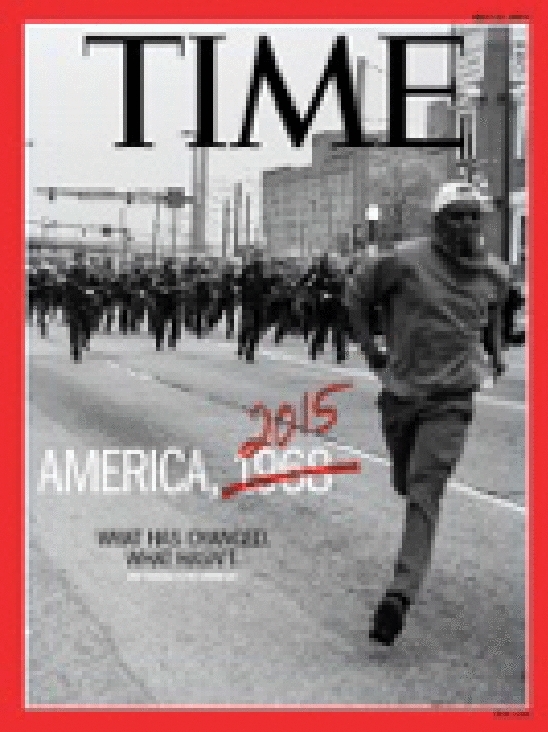
A *Time Magazine* cover featured by Devin Allen who took the photography during the Baltimore protests due to the aftermath of Freddie Gray’s death

Endorsed as “Word of the Year” in 2014 by the American Dialect Society, #BlackLivesMatter encapsulated how Black people utilized the online public space as a protesting venue. However, calling for justice by the Black community is far from the recent events. The racial discrimination and conflicts accounted for a major part of the early American history. As shown in the cover of *Time Magazine* of Fig. 1, the police brutality to Black people has been questioned whether it made any substantial progress from the Civil Rights Movement era in 1960s.

The unconstitutional violence by the state force produced constant victims of Black bodies and the Federal and local governments’ systemic failures of preventing excessive police violence to Black people gave no chance to seize human rights movement in continuation of the 1960’s Civil Rights Movement in the USA particularly within the Black communities. The applications of digital technologies drastically altered how protesters mobilize and take part in the movement. In the early morning on January 1, 2009, Oscar Grant, a 22-year-old Black father was shot and killed by Johannes Mehserle, a Bay Area Rapid Transit policeman, on the way home from the New Year’s Eve gathering with his friends. Numerous bystanders recorded the killing scene, and the mobile-phone videos were uploaded to *YouTube*, which recorded more than 500,000 views within four days after the incident. *KTVU*, a local news channel in Bay Area, broadcasted one of the raw footages and posted it on the media’s homepage, which recorded a thousand views per hour (Antony and Thomas 2010). The outraged protesters used Twitter to organize protests and inform citizens of the protest updates. Mehserle was charged with involuntary manslaughter, not second-degree murder, which fueled more resistance in the city of Oakland (Jackson et al. 2020). After Mehserle’s trial, Twitter was also employed to protect protesters in on-the-street protests from confrontations with police force using online technologies. Paulo Gerbaudo (2012) depicted this ICT-based coordination as, “a choreography of assembly…a process of symbolic construction of public space which facilitates and guides the physical assembling of a highly dispersed and individualized constituency” (p.5).

The protests and indignation against Grant’s wrongful death became a significant precursor to the BLM movement sparked by George Zimmerman’s acquittal from the murder of a seventeen-year-old Treyvon Martin (Dirks 2019). Protestors shared the hashtag #TreyvonMartin millions of times on Twitter while frequently accompanying #OscarGrant, #SeanBell, #EmmettTill, #FreddieGray, #WalterScott, #RenishaMcBride, and #ReikaBoyd, which are high profiling cases of Black body violence, next to #TreyvonMartin (Jackson et al. 2020). The second wave of the BLM movement was sparked by the death of eighteen-year-old Michael Brown murdered by Darren Wilson, a Ferguson policeman, on August 9, 2014. In 2013, Twitter enhanced its affordance allowing the display of uploaded pictures without clicking the source link. More than a couple of weeks after Michael’s death, people across the nation watched numerous images and smart-phone videos regarding the incident via various social media platforms. The BLM supporters applied the hashtags #Ferguson and #BlackLivesMatter to amass solidarity, to endorse resistance, to ascribe police brutality, to share protest strategies, to discuss specifically Ferguson, and to convey sentiments of the countermovement (Ince et al. 2017).

### Why #JusticeforBreonnaTaylor?

According to Jackson and her colleagues (2020), notable shifts were discovered in discourses and leaderships via the hashtag #TreyvonMartin Twitter networks. Not hundreds or thousands but millions of citizens shared smart phone-videos and images on Black Twitter and used some of them as a live footage to track how Treyvon was murdered in police custody (Hill et al. 2020, June 1). Scholars indicated the significance of “distributive framing” enabled via hashtags where problems and solutions were discussed within the non-hierarchical and decentralized networks (Bonila and Rosa 2015; Chong 2019a; Jackson et al. 2020). Though the BLM movement was established by Black women, public discourses often centered on the lives and deaths of Black men and boys at the hand of the state (Chatelain and Asoka 2015; Threadcraft 2017). Patton and Njoku (2021) said, “When attention is given to the violence against Black women, they are either blamed for their victimization or rendered invisible altogether. Black women’s labor as a driving force of the BLM movement is also largely unacknowledged” (p.1). Due the skewed attentions, people failed to form the broader understanding of police brutality and draw fair attention on police or state-sanctioned violence against Black women. Chatelain and Asoka (2015) addressed the following claims:

So far, the movement’s attention primarily to the experiences of black men has shaped our understanding of what constitutes police brutality, where it occurs, and how to address it. But black women—like Rekia Boyd, Michelle Cusseaux, Tanisha Anderson, Shelly Frey, Yvette Smith, Eleanor Bumpurs, and others—have also been killed, assaulted, and victimized by the police. Often, women are targeted in exactly the same ways as men—shootings, police stops, racial profiling. They also experience police violence in distinctly gendered ways, such as sexual harassment and sexual assault

(p. 54).

Both the extrajudicial deaths of Taylor and Floyd became the primary driver of the nationwide protests over racial injustice that erupted during the COVID-19 pandemic in the USA. (Oppel et al. 2020, May 30). Yet, there exist some differences between the two cases in view of the identity of the two victims. First, Breonna is a Black woman who has often battled with sexism, bigotry, and hate, i.e., #FastTailedGirls and misogynoir, and struggled with the stereotypical and objectified ways of portrayals on mass and digital media (Bailey and Trudy 2018). @FeministaJones, a Black feminist and writer, tweeted, “The Black Power movements (or whatever) are deeply rooted in supporting BM [Black Men] above all else, even those who abuse BW [Black Women] & girls #FastTailedGirls” (Jackson et al. 2020, pp.39–40). The African American Policy Forum launched by Kimberlé Crenshaw and Columbia Law School hosted a week-long webinar about Black women and girls’ status in the USA in 2015. During the conversation in the webinar, scholars and activists asserted that the systemic environment of state violence can only be completed when including investigations of Black women and girls’ racial injustice. By coining the term “intersectionality,” Crenshaw (1990) said, “many of the experiences of Black women face are not subsumed within the traditional boundaries of race or gender discrimination … cannot be captured wholly by looking at the women’s race or gender dimensions of those experience separately” (Crenshaw 1990, p. 1244). Therefore, it is important to examine how police violence against a Black woman was discussed in the #JusticeforBreonnaTaylor Twitter network.

Second, as Oscar Grant’s case, the police killing of Floyd was eye witnessed by many bystanders during the daytime and produced a large digital archive of citizen generated images and videos. However, Taylor’s case has a scarce chance to be witnessed by onlookers because she was shot and killed in her bedroom, a private space, and the police raid was executed shortly after midnight. While no comprehensive national data on police violence against Black women exists, many stories and statistics shared with the hashtag #sayhername disclose, “state-enabled violence against Black women can happen in less public spaces than that against Black men” (Chatelain and Asoka 2015; Jackson et al. 2020, p. 61). Third, the grand jury’s decision on her case made public on September 23, 2020, and no police officers were indicted directly from the killing of Breonna Taylor. Derek Chauvin, the ex-Minneapolis police officer who kneeled on George Floyd’s neck during his custody, was charged with second-degree murder, third-degree murder, and second-degree manslaughter by the jury on April 20, 2021, which made the first case of the white police officer’s conviction killing a Black person in Minnesota (Hayes et al. 2021, April 21).

In this study, I examined the #JusticeforBreonnaTaylor Twitter networks and analyzed discursive conversations and key players in the network. Additionally, I analyzed frequently accompanied hashtags next to #JusticeforBreonnaTaylor to survey the interconnected semantic representations via the hashtag. The concerned public were dissatisfied with and questioned the conclusion of the criminal proceedings because there were no homicide charges against the police officers involved in Breonna’s death, which occurred on September 23rd, 2020 (D1). The 15-h audio files of the grand jury’s investigations and inquiry, released on October 2nd (D2) by the public request, was also questionable and presented conflicting evidence against the conclusion of the criminal proceedings against the police officers. I applied a stage-based approach by independently investigating each network as before and after the release of the 15-h audio files and the following research questions were examined:

- RQ 1: Who are the key connectors in the #JusticforBreonnaTaylor Twitter networks in September and October 2020 respectively?
- RQ 2: What are the major themes shared in the #JusticforBreonnaTaylor Twitter networks in September and October 2020 respectively?
- RQ 3: What hashtags are frequently associated with #JusticeforBreonnaTaylor in the #JusticforBreonnaTaylor Twitter networks in September and October 2020 respectively?

## Methods

### Data collection

I retrieved tweets applying the search term #JusticeforBreonnaTaylor via Application Programming Interface (API) using “import from Twitter search network” function in NodeXL (Hansen et al. 2020). I acquired the datasets from September 1 to October 25 in 2020 and collected tweet relationships, including tweets, mentions, retweets, and replies-to, that included the hashtag #JusticeforBreonnaTaylor. A total of 631,176 tweets (edges) created by 101,200 Twitter users (vertices) were examined for this study. The September datasets consisted of 600,426 edges generated from 82,154 vertices and the October dataset comprised 30,750 edges created by 19,046 vertices. The data collection period of September dataset is from September 1 to September 30, 2020, which included September 23, 2020 when the grand jury’s decision on the Taylor case. October dataset was retrieved after the release of the 15-h audio files recorded the grand jury’s inquiry on October 2, 2020. The entire tweet information, including text, hashtags, images, and URLs, were retrieved in the data collection process. After removing duplicated edges, the final datasets comprised 82,359 and 25,106 unique edges for the September dataset (hereafter D1) and the October dataset (hereafter D2), respectively.

### Data analysis

To investigate the proposed research questions, this study employed a multi-method approach applying social network analysis, natural language processing, and qualitative examinations. To answer RQ1, I performed social network analysis focusing on centrality measurements by adopting betweenness centrality, page rank algorithm, eigenvector centrality, and in and out degree numbers conducted by NodeXL (Hansen et al. 2020). High centrality retains potential to affect actions and opinions of network participants because it could control information flow (Burt 1999). Betweenness centrality, which measures the shortest route between two other vertices of a given vertex, can often bridge gaps within the entire network (Hansen et al. 2020). This concept is closely related to weak ties that allow network participants to be exposed to critical information by boosting information-sharing as well as broadening the network participants’ information fields (Granovetter 1977, p. 50).

A high betweenness centrality vertex often become a hub that delivers a great deal of information traffic within the shortest paths qualifying it as a significant actor by developing discourses among various groups in the network (Easley and Kleinberg 2012). Thus, high betweenness centrality vertices are described as “top influencers” in the network compared to vertices simply retaining numerous followers. In and out degree numbers define the number of incoming and outgoing edges to a given vertex. Eigenvector centrality, dubbed as a prestige score, measures its linkage in a network. A high eigenvector centrality actor means that the vertex and its connected vertices demonstrated strong connectivity in a network (Negre et al. 2018). Page rank is a sister algorithm of the eigenvector centrality, which is often used to measure the popularity of website pages, such as Google.com (Austin 2006). In this study, betweenness centrality was employed as the principal measurement to define the key actors in addition to eigenvector centrality, page rank scores, and indegree and outdegree numbers.

To examine thematic characteristics of the discourse in the #JusticsforBreonnaTaylor Twitter network, I used Python to perform topic modeling by applying Latent Dirichlet Allocation (LDA) for D1 and BERTtopic modeling for D2 (RQ2). My original plan was applying BERTopic modeling for both D1 and D2, but despite multiple attempts, I could not complete BERTopic modeling for D1 analysis. Instead, I decided to apply LDA for D1 and discussed this issue in the Limitation section. I qualitatively examined information sharing behavior of the participants in the #JusticeforBreonnaTaylor Twitter network to further understand the conversational attributes. I used Python to extract and visualize popular hashtags in the network (RQ3). The Data cleaning process was conducted before topic modeling, which included removing emojis, non-textual expressions, URLs, and user indication with @ symbol, hashtags, retweets, and stop words, such as articles and non-content words. LDA topic modeling extracts topics via probability distributions and associations tested to all terms in the dictionary applying cues from context because it can associate words with pertinent meanings and avoid associating multi-meaning words (Blei et al. 2003; Topic Modeling 2016). For D2, I specifically applied BERTopic modeling that utilizes BERT embeddings and c-TF-IDF to generate dense clusters that enables easily translatable topics holding key words in topics (Grootendorst 2020). After processing BERT embeddings, UMAP (dimensionality reduction algorithm), and HDBSCAN (density-based algorithm) were used to create clustered documents (McInnes et al. 2017; McInnes et al. 2018). To extract topics from the clustered documents, class-based TF-IDF scores were calculated based on the formula below.

$$c - {\text{TF}} - {\text{IDF}}_{i} = \frac{{t_{i} }}{{w_{i} }} \times \log \frac{m}{{\sum\nolimits_{j}^{n} {t_{j} } }}$$

BERTopic modeling allows researchers to select diverse pre-trained embedding models and multiple n-gram models for topic representation. Pai (2022, June 23) claimed, “Pretrained word embeddings are the most powerful way of representing a text as they tend to capture the semantic and syntactic meaning of a word”. In this study, I selected three-gram analysis for D2 BERTopic modeling because it provided the most contextual information when compared to one-gram and two-gram analyses. To determine the best number of topics for D1 analysis, I surveyed lowest perplexity scores and highest coherence scores. To retrieve popular hashtags, I applied Panda library in Python and used online application (WordClouds.com) to create word clouds for D1 and D2.

## Results

- RQ 1: Who are the key connectors in the #JusticforBreonnaTaylor Twitter networks in September and October 2020 respectively?

Prioritized by the betweenness centrality measurement, Tables 1 and 2 presented the top 15 influencers in D1 and D2, respectively, based on betweenness centrality, page rank algorithm, in and out degree numbers, and eigenvector centrality. Among the top actors in D1, @mmpadellan having 916,300 followers, @funder having 987,200 followers, and @talbertswan having 171,900 followers were identified as social justice activists ranked as 1st, 2nd, 4th, and 13th Table 1. A Democratic party advocate organization having 477,800 followers (@thedemcoalition), the justice lawyer of the Breonna Taylor’s case (@attorneycrump), Attorney General of the Commonwealth of Kentucky (@kyoag and @danielcameronag), and Ben and Jerry’s ice cream company (@benandjerrys) were also identified as major actors in D1. In addition, ordinary Twitter users, such as @mania_tf, @mafuyuterus, @gauravvjw, @cipherequality, and @tpwkxclaire, played key roles to connect the participants in D1.

**Table 1:** The Top 15 Actors by Betweenness Centrality in the September #JusticeforBreonna Taylor Twitter Network

Rank	Vertex	Betweenness centrality	Page rank	Indegree	Outdegree	Eigenvector centrality
1	@mmpadellan	1,839,972,830.454	1516.360	4430	2	0.000
2	@funder	885,445,141.153	2022.107	6832	39	0.000
3	@mania_tf	427,484,867.940	33.206	0	174	0.000
4	@mafuyuterus	402,898,754.701	9.081	201	3	0.000
5	@thedemcoalition	371,760,427.338	930.443	3533	40	0.000
6	@attorneycrump	247,750,811.817	377.437	1318	4	0.000
7	@gauravvjw	239,646,288.174	5.521	1	45	0.000
8	@benandjerrys	216,431,941.806	410.202	930	1	0.000
9	@cipherequality	193,525,931.738	10.533	0	88	0.000
10	@tpwkxclaire	177,086,711.790	6.636	114	49	0.007
11	@kyoag	162,204,763.610	115.603	592	0	0.000
12	@_____a_nobody	161,688,527.899	2.389	3	51	0.000
13	@talbertswan	147,448,662.014	203.899	556	3	0.000
14	@danielcameronag	138,222,272.671	100.846	538	0	0.000
15	@ciara	121,283,114.645	381.524	854	1	0.000

**Table 2:** The Top 15 Actors by Betweenness Centrality in the October #JusticeforBreonnaTaylor Twitter Network

Rank	Vertex	Betweenness centrality	Page rank	Indegree	Outdegree	Eigenvector centrality
1	@attorneycrump	52,941,663.781	551.630	1612	3	0.019
2	@sifill_ldf	33,826,174.078	427.205	1206	4	0.003
3	@blackwomenviews	25,334,922.421	404.754	986	1	0.001
4	@violadavis	24,364,509.856	361.312	836	1	0.000
5	@kyoag	23,654,854.026	163.243	637	0	0.002
6	@pettylupone	19,274,140.987	284.552	673	1	0.000
7	@atticascott4ky	14,008,236.991	161.347	500	14	0.000
8	@complexsports	13,775,545.675	244.379	542	1	0.000
9	@jifueko (suspended)	9,914,986.931	179.052	400	1	0.000
10	@danielcameronag	9,259,312.295	59.023	241	0	0.000
11	@karebearkisses	8,857,017.924	11.554	2	44	0.001
12	@uniqueloves	8,717,712.958	13.781	9	48	0.001
13	@_winston_chow	8,314,829.196	10.585	8	44	0.001
14	@queenvlion1	6,681,056.136	9.544	2	31	0.001
15	@talbertswan	5,084,502.829	42.220	143	4	0.000

As shown in Table 2, several top actors in D1, such as @attorneycrump, @kyoag, @danielcameronag, and @talbertswan, were highly ranked in D2. In addition, @Sifill_LDF (President & Director-Counsel of NAACP Legal Defense and Educational Fund) having 330,000 followers, described as the nation's premier civil rights law organization, was the second top influencer in D2. The founder of BlackWomenViews Media (@blackwomenviews) and the Kentucky House of Representative Attica Scott (@atticascott4ky) were also identified as key connectors in D2. Sports and culture media (@complexsports) having 413,500 followers and Viola Davis (@violadavis), an African-American actor and producer who achieved Triple Crown of Acting were identified as top influencers. Social justice and anti-racism activists, such as @uniqueloves and @_winston_chow, were also highly ranked, and ordinary users, i.e. @karebearkisses and @queenvlion1, were also recognized as major actors in D2.

**Fig. 2:**
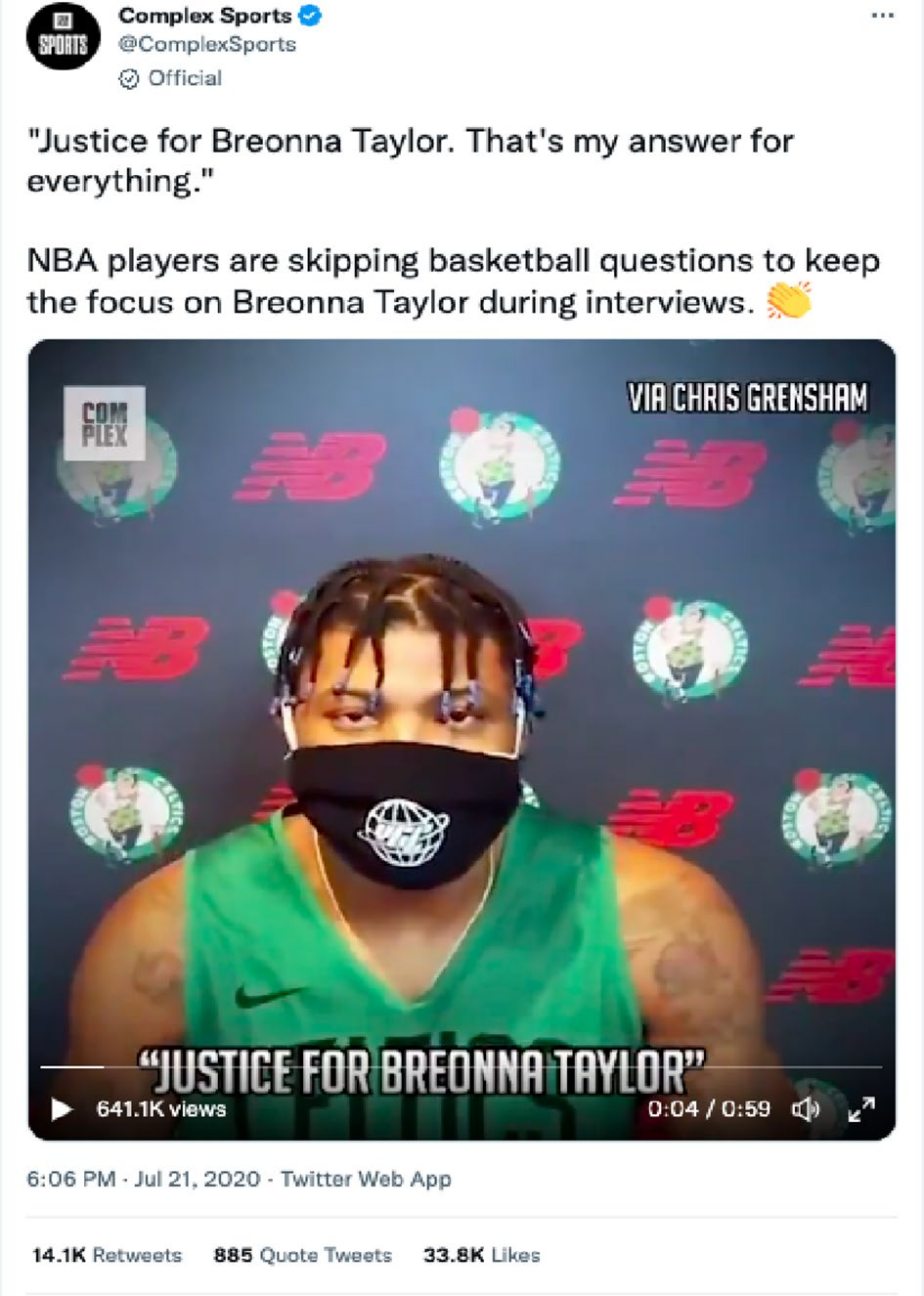
A tweet posted by @ComplexSports

The important stakeholders of Breonna Taylor’s legal case, a civil rights and Taylor’s trial lawyer Benjamin Crump and Kentucky Attorney General Danial Cameron, were the outstanding and only key connectors detected in both D1 and D2 networks excluding @talberswan. Among public accounts, Ben and jerry’s ice cream company (@benandjerrys) and Complex.com (@complexsports) were identified as top influencers. The two companies presented solidarity to bring justice to Taylor’s killing. For example, on September 23, @benandjerris posted a tweet saying, “No Justice. No Peace. #JusticeForBreonnaTaylor” and another tweet sharing a web petition link with a statement saying, “By failing to bring murder charges in the brutal killing of Breonna Taylor, Attorney General Cameron has failed to deliver justice for Bre” (colorofchange.org). Complex.com is a sports and culture media outlet, and @complexsports posted a tweet about NBA players’ efforts to bring attention to and support for the Taylor’s case in the court (See Fig. 2). The most remarkable change from D1 to D2 was the role of Black woman activist, celebrity, and politician, as highlighted in yellow in Table 2, to connect the #JustforBreonnaTaylor network.

- RQ 2: What are the major themes shared in the #JusticforBreonnaTaylor Twitter networks in September and October 2020 respectively?

Extracted key words next to probability scores from topic modeling are displayed in Tables 3 and 4. The higher probability scores illustrate the higher chances of keywords to be included in the assigned topic id. The strengths of LDA-based topic modeling are 1) connecting words with related meanings and separate uses of words from multiple meanings by utilizing clues from the context 2) subjective bias in analyzing data is minimized due to mathematical algorithm (Diesner et al. 2015). As illustrated in Table 3, topics of D1 were about demanding justice for the Taylor’s “murder” case (topic id 6 and 10) and protect black women’s life (topic id 8 and 9). The participants of D1 demanded that the white policemen must be charged (topic id 3) and support for right actions and donations (topic id 7,8, and 10) while urging protests and voting for the 46th Presidential election (topic id 1, 6, and 10). The most eminent theme was to encourage people to sign a petition asking to resubmit the Taylor’s case under a special prosecutor (topic id 1,6,7, and 10) after the announcement on September 23th made by KY AG Cameron that no involved officers of the incident were charged directly to her death (Legal Defense Fund 2022, August 4). Black Twitter viewed the event is as “violence against race/ racism” (topic id 9) and presented a lack of confidence to the jury (topic id 9).

**Table 3:** Top 10 topics extracted by LDA from D1

Topic id	Terms
1	'0.891*"go" + 0.044*"important" + 0.006*"happen" + 0.002*"vote" + ' '0.001*"viral" + 0.007*"tag" + 0.012*"sure" + 0.019*"confirm" + ' '0.001*"already" + 0.004*"signature"
2	0.799*"could" + 0.080*"rest" + 0.003*"jury" + 0.001*"least" + ''0.050*"believe" + 0.020*"freedom" + 0.003*"sorry" + 0.067*"late" + ''0.056*"care" + 0.058*"enjoy"
3	'0.757*"say" + 0.093*"name" + 0.060*"police" + 0.031*"take" + 0.009*"white" '' + 0.005*"also" + 0.004*"charge" + 0.410*"forget" + 0.009*"keep" + ''0.069*"must"'
4	0.649*"still" + 0.340*"fact" + 0.122*"walk" + 0.345*"freely" + 0.001*"head" '' + 0.060*"wrap" + 0.001*"tag" + 0.020*"trend" + 0.560*"think" + ''0.001*"system"'
5	0.653*"damn" + 0.061*"next" + 0.022*"end" + 0.018*"money" + 0.013*"learn" + ''0.004*"level" + 0.003*"protest" + 0.001*"war" + 0.001*"drug" + 0.001*"low"'
6	0.584*"break" + 0.180*"justice" + 0.129*"hope" + 0.069*"petition" + ''0.028*"help" + 0.001*"sue" + 0.001*"department" + 0.007*"give" + ''0.004*"chain" + 0.090*"know"'
7	0.574*"want" + 0.104*"get" + 0.160*"many" + 0.090*"brutality" + ''0.009*"donate" + 0.210*"sign" + 0.321*"spread" + 0.001"word" + ''0.089*"tweet" + 0.001*"pressure"'
8	0.433*"black" + 0.149*"let" + 0.128*"woman" + 0.078*"life" + 0.010*"fight" '' + 0.001*"stand" + 0.200*"protect" + 0.0049*"player" + 0.002*"soccer" + ''0.080*"deserve"'
9	0.265*"cause" + 0.120*"peace" + 0.111*"heart" + 0.030*"violence" + ''0.009*"remember" + 0.0560*"racism" + 0.090*"race" + 0.070*"search" + ''0.077*"healing" + 0.050*"book"'
10	0.232*"action" + 0.152*"right" + 0.105*"murder" + 0.009*"support" + ''0.001*"tag" + 0.060*"donate" + 0.100*"show" + 0.030*"twice" + 0.060*"sign" '' + 0.001*"instead"'

**Table 4:** Top 20 Topic Extracted with Three Grams by BERTopic Modeling in D2

Topic id	Count	Terms
63	989	'as shield for'*0.017 + 'decided the case'*0.017 + 'had already made'*0.017 + 'shield for decision'*0.017 + 'already made he'* + 0.017 + 'jury as shield'*0.017
14	843	'much from blaming'*0.036 + 'her death really'*0.036 + 'tactics incompetence that*0.036 + 'need for police'*0.036 + 'his department tactics'*0.036 + 'incompetence that her'*0.036 + 'blaming the walker'*0.036
9	269	'who commit the'*0.085 + 'ballistics don support'*0.084 + 'write the ballistics'*0.085 + 'the ballistics don'*0.085 + 'ballistics don*0.084
56	251	'video credit apanetwork'*0.049 + 'since her murder'*0.049 + 'continue to video'*0.049 + 'murder and justice'*0.049 + 'to video credit'*0.049 + 'until then we'*0.049
58	247	'on the tragic'*0.052 + 'the anger and'*0.052 + 'care of black'*0.052 + 'understand the anger'*0.052 + 'the tragic shooting'*0.052 + 'righteous indignation that'*0.052
90	196	'mistaken if they'*0.043 + 'why the release'*0.043 + 'all about cover'*0.043 + 'about cover they'*0.043 + 'not vote out'*0.043 + 'they re mistaken'*0.043 + 'cover they re'*0.043
106	184	'and her murderer'*0.063 + 'murderer brett is*'0.063 + 'people murder are'*0.063 + 'her murderer brett'* 0.063 + 'being on bond'*0.053 + 'sense people murder'*0.052
128	157	'supremacy sycophantic pathetic'*0.032 + 'allow white'*0.032 + 'murder the grand'*0.032 + 'white supremacy sycophantic'*0.032 + 'dancing white supremacy'*0.032 + 'sycophantic pathetic excuse'*0.032 + 'buck dancing white'*0.032
138	81	'week episode is'*0.06 + 'cover the murder'*0.06 + 'murder of keep'*0.06 + 'this week episode'*0.06 + 'keep lookout for'*0.06
511	65	'the brutal murder'*0.057 + 'indict white for'*0.036 + 'racism being dead'*0.036 + 'white jury two'*0.036 + 'jury two white'*0.036 + 'indict white', 0.036 + 'all white jury'*0.036

Due to release of the audio file recorded the grand jury’s inquiry on October 2, 2020, the public had a clearer picture on how the case was ruled. When compared to D1, top topics of D2 presented strong criticism on flaws and bias made during the judicial process (topic id 63, 14, 90) while expressing powerful sentiments of indignation stating, “the brutal murder” and “her murderer” (topic id 58, 106, 128, 511). Particularly, many topics included sentiments of distrust on the ruling procedure, systemic failure of the KY’s policing system, and KY AG Cameron (topic id 9, 14, 63, 56, 138, 90, 511). Like D1, the most shared topics was demanding justice for the case by properly investigating the case and prosecuting the police officers involved with killing Breonna Taylor mentioning, “indite white for” (topic id 56, 90, 511).

As presented in Table 4, top topics in D2 criticized the judicial process of the Taylor’s case arguing that it was used to protect the powerful from responsibility (topic id 63, 90, 138, and 511). For example, a tweet said, “Too much of the law is a smokescreen to use process as a shield. The goal is to protect the powerful from accountability. This just affirms what we already knew.” Many #JusticeforBreonnaTaylor network participants expressed indignation about police tactics that resulted in Taylor’s death (topic id 14). For example a tweet wrote, “So much BS from Mattingly blaming the victims (Breonna & boyfriend Kenneth Walker) for her death! Really it was his department’s policies, tactics & incompetence that killed her. There was no need for police to go to her apartment in the 1st place.” Many people described Taylor’s death as a (brutal) murder case (topic id 56, 106, and 511) and demonstrated strong interests about how evidence was managed by the authorities, such as the ballistics report and ethnic background of jury (topic id 9, 56, and 511). For example, a tweet stated, “@DanielCameronAG So the Ballistics report do not support your claims Kenneth, Breonna’s boyfriend shot an officer. Your license needs to be revoked and you should never practice law again. #Coward #Sellout #Liar.” Especially, many people were outraged by KY Attorney General Danial Cameron’s misinterpretation on the findings of the grand jury (topic id 63, 90, 128, 138, and 511). For example, a tweet said, “KY AG @djaycameron never sought charges for #BreonnaTaylor’s murder. The grand jury process was a farce, a tool used by a buck dancing, white supremacy apologizing, sycophantic, pathetic excuse of a Black man to allow white cops to get away with murder.”

**Fig. 3:**
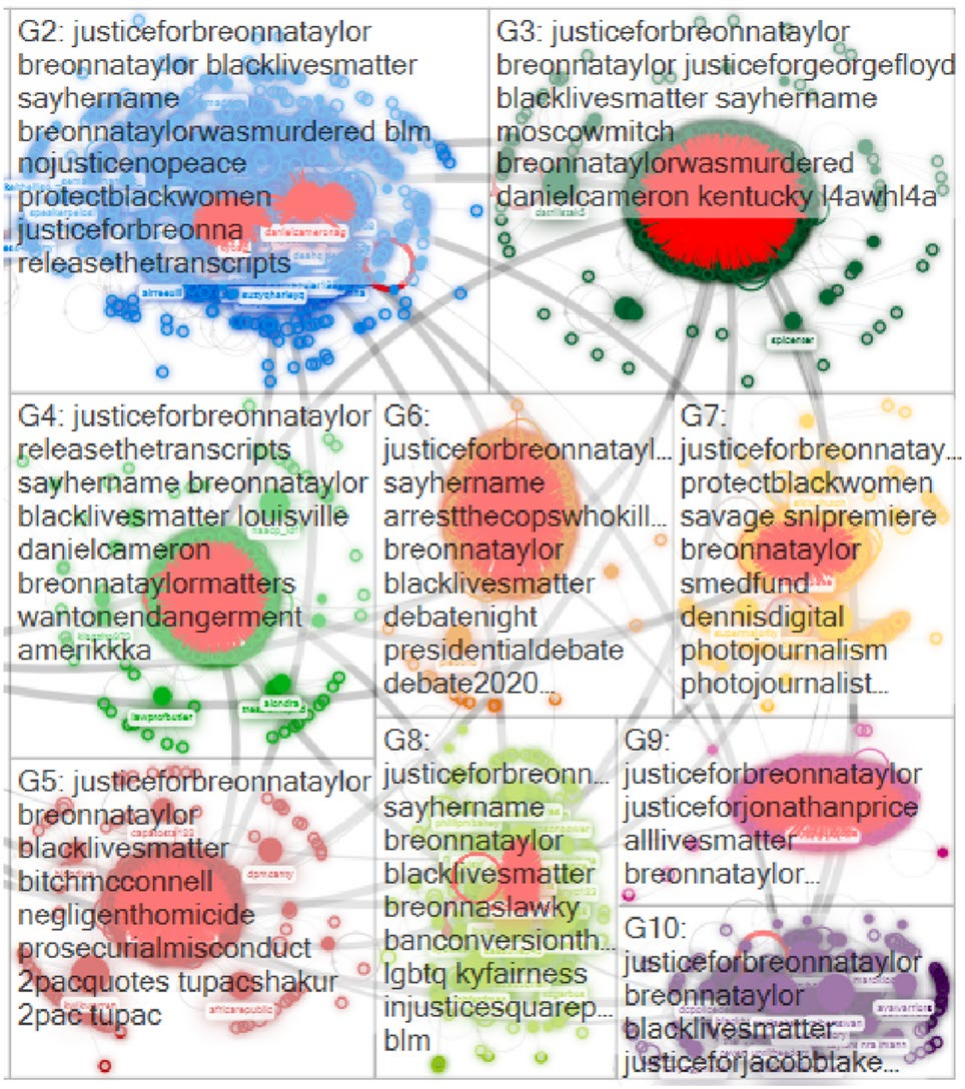
Top ten clusters in D2 with the most frequently shared words in each group

These top topics were evident through largely shared words in D2. Figure 3 illustrates the top ten groups in D2 applying the Clauset–Newman–Moore cluster algorithm to create the clusters and graphed using the Harel–Koren Fast Multiscale layout algorithm, which is extensively adopted in communication studies to measure information flow (Clauset et al. 2004). The network graph demonstrated the characteristics of “community clusters,” which comprise multiple small- and medium-sized conversational clusters instead of one dominant cluster according to interests and relevance of each cluster regarding the event with limited impact from top influencers compared to other discourse archetypes on Twitter, i.e., broadcast and polarized crowd networks (Smith et al. 2015). The most frequently shared words in each cluster represented similar themes with the BERTopic analysis of D2 (Table 4). For example, breonnataylor was murdered (G2), arrest the cop who killed Breonna Taylor (G6), negligent homicide (G5), and prosecurial misconduct (G5) appeared as largely shared in D2 and Danial Cameron was frequently mentioned in the conversation as well (G3 and G4).

On top of that, release the (court) transcripts (G3 and G4) and wanton endangerment (G4) displayed the participants’ attention regarding legal charges to the Louisville policemen who were involved with the botched raid to Taylor’s apartment. Racism behind the event was displayed with amerikkka (G4) and particularly participants in G6 focused on the US 2020 presidential election by largely sharing debate night, presidential debate, debate 2020. Unlikely with the BERTopic modeling, blacklivesmatter (G2, G3, G4, G5, G6, G8, and G10) and sayhername (G2, G3, G4, G6, and G8) extensively appeared in many clusters. This is because hashtags were included to analyze top words. In Fig. 3, the connected edges with top ten influencers from Table 2 were indicated in red, which demonstrated their central roles to link and sustain each cluster as well as the entire group in terms of network typology.

- RQ 3: What hashtags are frequently associated with #JusticeforBreonnaTaylor in the #JusticforBreonnaTaylor Twitter networks in September and October 2020 respectively?

As a community-oriented practice and bolstering folksonomy, Twitter users create and share hashtags to organize and disseminate information, agendas, feelings, or opinions about targeted events or news, which are accompanied by the # symbol and eventually applied to draw attention (Chong 2019b; Wang et al. 2011 October). In D1, hashtags about demanding justice for Black victims who killed by police brutality or in police custody were largely distributed, such as #justiceforjacobblake, #justiceforgeorgefloyd, #nojusticenopeace, #justiceforelijahmcclain, and #justiceforauntytanyaday. Protests to bring justice to the Taylor case were also organized and promoted via hashtags, i.e., #noderby146 (rank 8) and #portlandprotests (rank 23) (Table 5).

**Table 5:** Top 30 Hashtags Accompanied by #JusticeforBreonnaTaylor in September Twitter Network

Rank	Hashtags	Count	Rank	Hashtags	Count	Rank	Hashtags	Count
1	Justiceforbreonnataylor	178,773	11	socialistanyday justiceforjacobblake	522	21	wantonendangerment louisville	194
2	Justiceforjacobblake justiceforjacobblake nojusticenopeace	10,628 (128)	12	breakingnews anonymous legion expectus anons anonops anonymousforthevoiceless justiceforjacobblake justiceforall	436	22	portlandprotests breonnataylormatters breonnataylor blm pdx blacklivesmatter portland	188
3	breonnataylor	4215	13	justiceforjacobblake justiceforelijahmcclain	360	23	dictatortrump amerikkka trumpcoupplot breonnataylormatters	169
4	blacklivesmatter justiceforgeorgefloyd saytheirnames	2399	14	resist wearamask vote	303	24	votehimout votehimout2020 votebluetoendthis nightmare blacklivesmatter	155
5	breaking breakingnews whistleblowers	2089	15	breonna chargemylecosgrovewithmurder chargebretthankisonwithmurder chargejonathanmattinglywith murder	301	25	nojusticenopeace	147
6	blacklivesmatter blacklivesstillmatter	2134 (279)	16	blm sayhername	271	26	nooneisabovethelaw sayhername onev1 demvoice1	145
7	sayhername	1794	17	tbt blacklivesmatter stayme7o	217	27	blacklivesmatter voteoutracism	140
8	noderby146 (noderby146 nojusticenoderby)	576 (160)	18	detroityouthchoir kidjay glory weareone unity peace	212	28	breonnataylormatters	139
9	sayhername blacklivesmatter	573	19	justiceforjacobblake vmas vmas vmas2020	200	29	istillcantbreathe	133
10	justiceforjacobblake blacklivesmatter	557	20	justiceforauntytanyaday justiceforwalker blacklivesmatter	197	30	stoppolicebrutality voteoutthegop biden2020 traitorssupport traitortrump votelikeyourlife dependsonit	105

**Fig. 4:**
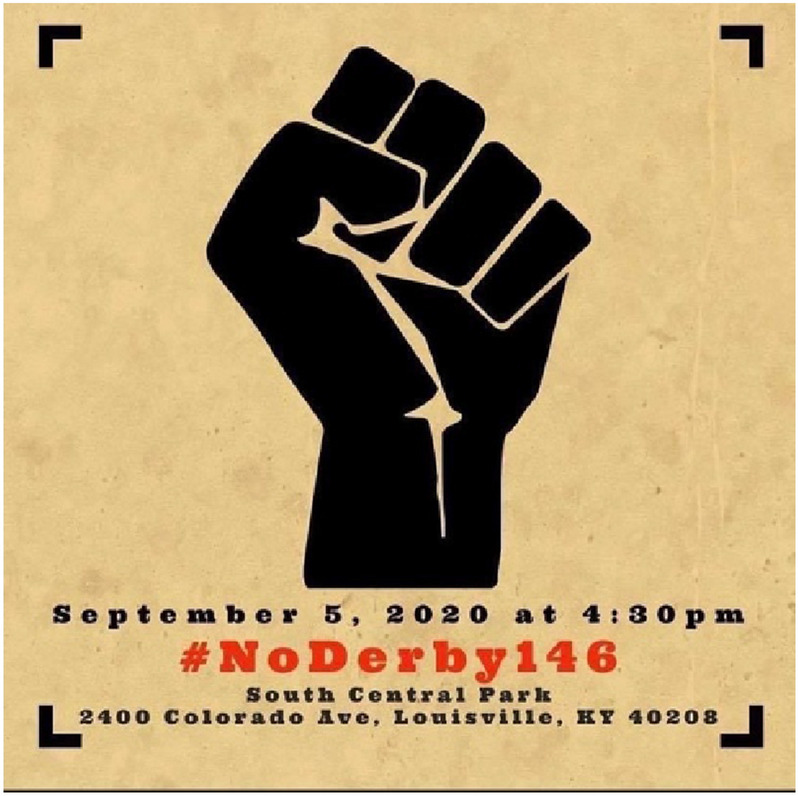
A digital flyer of the NoDerby146 protest

**Fig. 5:**
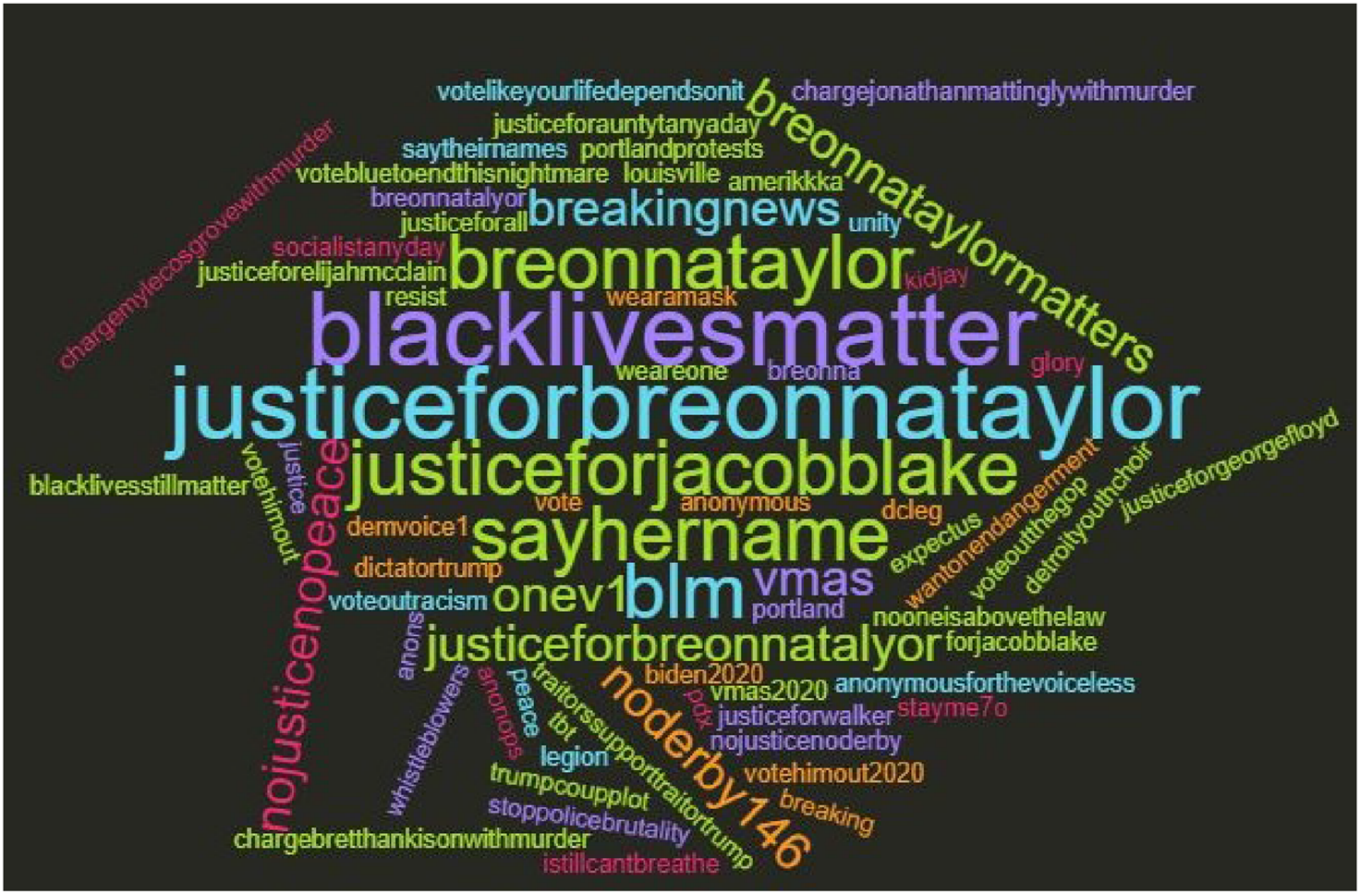
A word cloud generated based on top 30 hashtags in D1

As presented in Fig. 4, the NoDerby146 rally was organized to demand delayed justice to the Taylor case and especially call on the three policemen involved in her shooting to be charged in the event day of the Kentucky Derby, which is an annual horse race held in Louisville, Kentucky. The name of the three cops were also largely shared and promoted as hashtags, i.e., #chargemylecosgrovewithmurder, #chargebretthankisonwithmurder, #chargejonathan.mattinglywithmurder (rank15) in D1. The hashtags #blacklivesmatter and #sayhername were presented as top hashtags (rank 4, 6, 7, 9, 10, 16, 17, 18, 21, 23, 25, 27, and 28). Figure 5 visualizes the top 30 hashtags as a word cloud where the size of the hashtags represented the frequency of the hashtags appeared in D1.

Table 6 presents the top 30 hashtags associated with #JusticeforBreonnaTaylor in D2. Similar to the largely shared hashtags in D1, #blacklivesmatter and #sayhername (rank 3, 4, 5, 6, 7, 8, 15, 19, 26, 27, 28, and 29) as well as hashtags demanding justice for victims of police brutality (rank 8, 9, 12, 14, 21, and 26) were identified as top hashtags in D2. As previously examined in top words analysis in Fig. 3, a hashtag about demanding a court action, i.e., #releasethetranscripts (rank 10), was largely shared. Particularly, Black women were honored via the hashtags #protectblackwomen (rank 6) and #blackwomenaredivine (rank 9), and especially names of Black women killed by the authorities were largely distributed in D2 (rank 9). Distrust to Danial Cameron was strongly revealed via hashtags, i.e., #agcameroniscorrupt (rank 16) and #coverup (rank 20). As close to the 46th presidential election day on November 3rd, 2020, hashtags about encouraging the participants to vote for the Democratic presidential candidate appeared as one of the top hashtags in D2 (rank 11 and 18).

**Table 6:** Top 30 Hashtags Accompanied by #JusticeforBreonnaTaylor in October Twitter Network

Rank	Hashtags	Count	Rank	Hashtags	Count	Rank	Hashtags	Count
1	Justiceforbreonnataylor	13,161	11	Votehimout votehimout2020 votebluetoendthisnightmare blacklivesmatter	100	21	Banconversiontherapy lgbtq kyfairness	63
2	Breonnataylor	2533	12	Justiceforgeorgefloyd justiceforahmaudarbery justiceformanuelellis	96	22	Breonnataylor demvoice1	58
3	Blacklivesmatter (us Blacklivesmatter)	858 (274)	13	Breonnataylor onev1	85		Blmprotest	58
4	Sayhername arrestthecopswhokilled breonnataylor	866	14	Justiceforjonathanprice justiceforelijahmcclain blm	81	24	Breonnaslawky	57
5	Sayhername sayhername breonnataylor	541 (321)	15	Breonnataylor sayhername blacklivesmatter	78	25	Wantonendangerment louisville	54
6	Protectblackwomen savage snlpremiere	673	16	Agcameroniscorrupt	73	26	Blacklivesmatter justiceforgeorgefloyd	47
7	Blacklivesmatter saytheirnames sayhername breonnataylor	195	17	Endqualifiedimmunity	67	27	Sayhername alphaphialpha apa1906network forthe7jewels sayhername breonnataylor	45
8	Justiceforgeorgefloyd justiceforkendrickjohnson blacklivesmatter (justiceforgeorgefloyd justiceforkendrickjohnson justiceforahmaudarbery)	98 (90)	18	Breonnataylor eleanorbumpers voteforbreonna millionmanmarch25	66	28	Blacklivesmatter UKisnotinnocent	44
9	Blacklivesmatter blackwomenaredivine justiceforregiskorchinskipaquet mariellepresente layleenxtravaganzapolanco wakeishawilson sandrabland amlesethaile sumayadalmar sayhername	107	19	Blacklivesmatter breonnataylorwasmurdered	65	29	Breatheact defundpolice sayhername breatheday	44
10	Releasethetranscripts	102	20	Coverup	64	30	The source	44

**Fig. 6:**
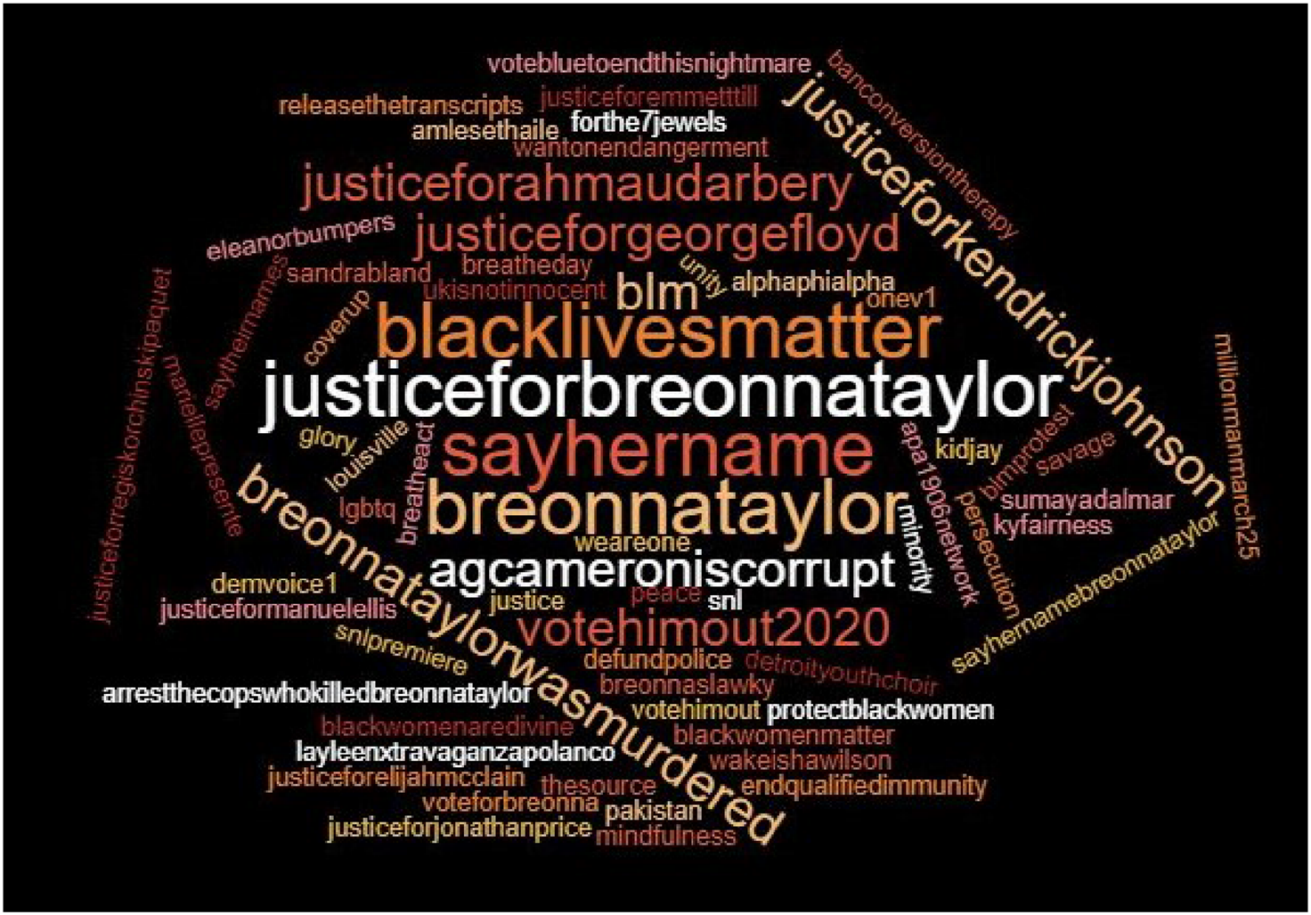
A word cloud generated based on top 30 hashtags in D2

Figure 6 visualizes the top 30 hashtags as a word cloud and the size of the hashtags represent the frequency of the hashtags appeared in D2.

One of the most outstanding characteristics in the application of hashtags was remembering and honoring those numerous Black victims by police brutality in the USA and demanding justice via prosecution of criminals using the hashtag #Justfor next to each victim’s name. Allyship was also expressed via hashtags, i.e., #UKisnotinnocent (rank 28 in Table 6) and #Justiceforauntytanyaday (rank 20 in Table 5), which extended the realm of the Black Lives Matter Movement as the global manifesto as many leading activists of the #StopAsianHate and #HongKongProtests movements expressed supports and inspirations from the Black Lives Matter Movement.

## Conclusions and discussion

Taking a stage-based approach, this study examined Twitter networks with particular focus on key actors and thematic analysis of textual tweets, and hashtags comparing two #JusticeforBreonnaTaylor Twitter networks. In both September and October networks, mainly social justice activists and some ordinary participants were commonly identified as top influencers and key actors. Reflecting the network participants’ attention to the criminal proceedings of the Taylor’s case, Benjamin Crump (@attroneycrump) was identified as the top connector in the October network while the rank of Danial Cameron (@kyoag) also moved up to 5th in D2. Crump and Cameron are the key individuals of the legal procedure of the Taylor’s case and the network participants’ foci to their messages on Twitter illustrated the public’s strong interests to both the legal actions and decisions on this case.

The study findings evidently show activism and support for Black women, and it is notable that half of the top connectors in D2 were black women while their presence was mediocre in D1. The hashtag #sayhername, an instructional and policy-oriented, focused on Black women victims by police violence and shared nationwide and communal attacks against Black women. Black feminist scholars claimed that Black women victims of the state violence were overwhelmingly described and understood their relationships with Black men victims by state violence (Jackson et al. 2020, p. 52). However, when Sandra Bland died in a jail in Texas under a police custody in July 2015, the hashtag #sayhername reached a breaking point in visibility along with her hashtagified name #SandraBland and #justiceforSandy. Black Twitter scholars argued that #sayhername is conceptually and circumlocutorily linked with the overall Black Lives Matter movement because the hashtag has often shared on Twitter next to #BlackLivesMatter. The findings of this study confirmed that the most frequently shared hashtags were #justiceforBreonnataylor, #Breonnataylor, #blacklivematter, #sayhername, and #arrestthecopswhokilled.

The thematic analysis via topic modeling, top words, and hashtags demonstrated that.

The participants in both #JusticforBreonnaTaylor Twitter networks strongly demanded righteous legal prosecution to the three Louisville cops that involved in the botched raid of Taylor’s apartment. Many participants expressed distrust on Danial Cameron’s involvement in the legal case. Multiple topics suggested that Cameron breached the public trust and failed to comply with his duties by misguiding the grand jury of the Taylor’s case. One tweet said, “People need to be held accountable for the incorrect information used during the trial.” Three grand jurors of the Taylor case filed the petition to impeach Cameron because “He lied about what was presented to the grand jurors, he lied about what options were given to the grand jurors and he lied about the decisions the grand jury made” (Wise 2021, January 21).

Black Americans have been the largest racial demographic group among all Twitter users in the USA (Pew Research Center 2022, November 16). This study ascertained that the #JusticeforBreonnaTalyor Twitter network as another community of Black Twitter. The findings of the study discovered that the participants not only shared breaking news and important information but also organized protests and tagged people to spread messages about the Taylor’s case on Twitter. It is important to note that the network participants purposefully utilized textual information and hashtags in their tweets. The participants conversed the major issues about the Taylor’s case and set the agendas for the next action, such as encouraging to vote for then the upcoming presidential election.

In the cases of past police violence against the Black bodies, #JusticeforOscarGrant and #JusticeforTreyvonMartin were created and shared but #OscarGrant and #TreyvonMartion (names of victims) or #Ferguson and #FalconHeights (locations of the events) were more largely diffused. However, after George Floyd’s death in May 2020, #justiceforGeorgeFloyd became the top trending hashtag followed by many other victims, including #JusticeforJacobBlake, #JusticeforDaunteWright, #JusticeforDanialPrude, and #JusticeforBreonnaTaylor. This suggests that people on Black Twitter collectively developed and shared their agenda that specified “demanding judicial justice to the state-sanctioned murderers” from the broad theme of “Black Lives Matter.” As the self-explanatory hashtag, the strong and clear focus of the #JusticeforBreonnaTalyor Twitter network was demanding justice for the Breonna Taylor’s death. The history of Black victims by state violence were resonated via topics and hashtags in the network. Black women were substantially contributed to the visibility of the #JusticeforBreonnaTalyor hashtag constructing political coalitions.

This study is not free from its own limitation. The major limitation is that the findings resulted from a limited datasets collected Twitter search API. The maximum number of tweets collected via NodeXL Pro restricts around 18,000 tweets per retrieval. Thus, like most social media studies, the findings of this study low generalizability. This study employed two different topic modeling algorithms, Bertopic and LDA, and how this implementation affected on data analysis process is not fully accessed.

In the future study, I would like to adopt the second coder to define topics from topic modeling applying inter coder reliability measurement. I think this step could enhance to the subjective part of topic modeling analysis. I also plan to analyze #justiceforgeorgefloyed Twitter networks, and it will be interesting to compare any similarities and differences with the findings of this study in terms of key actors and top topics.
